# A novel *in vitro* cell model of the proteinase/antiproteinase balance observed in alpha-1 antitrypsin deficiency

**DOI:** 10.3389/fphar.2024.1421598

**Published:** 2024-07-01

**Authors:** Celine H. Chen, Helena Crisford, Aaron Scott, Elizabeth Sapey, Robert A. Stockley

**Affiliations:** ^1^ Acute Care Research Group, Institute of Inflammation and Ageing, Queen Elizabeth Hospital Birmingham, University of Birmingham, Birmingham, United Kingdom; ^2^ National Institute for Health and Care Research (NIHR) Birmingham Biomedical Research Centre, Institute of Translational Medicine, Birmingham, United Kingdom; ^3^ Centre for Translational Inflammation Research, Queen Elizabeth Hospital Birmingham, University of Birmingham, Birmingham, United Kingdom; ^4^ Department of Sleep and Lung Function, Queen Elizabeth Hospital Birmingham, Birmingham, United Kingdom

**Keywords:** neutrophil elastase, proteinase 3, proof of concept, MPH966, alpha-1 antitrypsin deficiency

## Abstract

**Background:** Alpha-1 antitrypsin deficiency (AATD) is a genetic condition resulting from mutations in the alpha-1 antitrypsin (AAT) protein, a major systemic antiproteinase, resulting in reduced/no release of AAT, disrupting the proteinase/antiproteinase balance. A sustained imbalance can cause structural changes to the lung parenchyma, leading to emphysema. Predicting and assessing human responses to potential therapeutic candidates from preclinical animal studies have been challenging. Our aims were to develop a more physiologically relevant *in vitro* model of the proteinase/antiproteinase balance and assess whether the data generated could better predict the efficacy of pharmacological candidates to inform decisions on clinical trials, together with expected biomarker responses.

**Methods:** We developed an *in vitro* model assessing the proteinase/antiproteinase balance by the changes in the fibrinogen cleavage products of neutrophil elastase (NE) and proteinase 3 (PR3). This allowed the assessment of physiological and pharmaceutical neutrophil serine proteinase (NSP) inhibitors to determine the putative threshold at which the maximal effect is achieved.

**Results:** AAT significantly reduced NE and PR3 activity footprints, with the maximal reduction achieved at concentrations above 10 μM. The inhibitor MPH966 alone also significantly reduced NE footprint generation in a concentration-dependent manner, leveling out above 100 nM but had no effect on the PR3 footprint. At levels of AAT consistent with AATD, MPH966 had an additive effect, reducing the NE activity footprint more than either inhibitor alone.

**Conclusion:** Our results support an inhibitor threshold above which the activity footprint generation appears resistant to increasing dosage. Our model can support the testing of inhibitors, confirming activity biomarkers as indicators of likely pharmaceutical efficacy, the assessment of NSP activity in the pathophysiology of emphysema, and the likely function of biological or pharmacological inhibitors in disease management.

## 1 Introduction

Alpha-1 antitrypsin deficiency (AATD) is an inflammatory condition arising from genetic mutations in the alpha-1 antitrypsin (AAT) gene SERPINA1 (Zorzetto et al., 2008), resulting in little/no secretion of the major antiproteinase AAT that is crucial for the modulation of the destructive potential of neutrophil serine proteinases (NSPs). AAT is a polymorphic protein encoded on chromosome 14 expressed by two alleles in a codominant manner (Sefton et al., 1990). The deficiency severity varies depending on the homozygous or heterozygous combination of the normal or deficient alleles.

Most healthy individuals carry two M alleles, resulting in a plasma concentration of 20–40 μM (Brantly et al., 1991; Ferrarotti et al., 2012). The most common deficiency alleles associated with the reduced production of AAT are the S and Z alleles. The S variant results from a point mutation substituting glutamic acid with valine at position 264 (rs17580) (Long et al., 1984). The S variant produces 40%–50% less AAT compared to the normal M variant and shows an increased likelihood of polymerization (Elliott et al., 1996). However, the S variant on its own is less concerning from a clinical viewpoint unless combined with the Z variant, which results in a plasma AAT level between 8 and 15 μM, breaching the threshold of adequate protection against NSPs (<11 μM) (Wewers et al., 1987; Brantly et al., 1991; Ferrarotti et al., 2012). The most prevalent and clinically relevant deficient cases of AATD are those homozygous for the Z allele, caused by a point mutation replacing glutamic acid to lysine at position 342 (rs28929474) in the mature AAT protein (Kidd et al., 1983). The Z-variant AAT (Z-AAT) protein is much more susceptible to polymerization and formation of intracellular aggregates, which impedes secretion into the circulation, resulting in very low plasma AAT levels of less than 5 μM (Bathurst et al., 1984; Loebermann et al., 1984; Cox et al., 1986). Additionally, Z-AAT also displays a reduced association rate with target serine proteinases, indicating that they are less effective than the normal AAT protein in inhibiting serine proteinases (Ogushi et al., 1987).

Homeostasis is maintained between proteinases and protective antiproteinases in healthy tissues. However, this homeostasis is markedly disrupted in AATD, resulting in a pathophysiological proteinase/antiproteinase imbalance (Stockley, 1999; Sinden and Stockley, 2013; Sinden et al., 2015). The reduced amount of AAT disrupts this physiological homeostasis, resulting in enhanced proteinase activity, especially in the airways, causing irreversible emphysematous and airway changes associated with chronic obstructive pulmonary disease (COPD) (Stockley, 1999). Uninhibited proteinases are able to cleave major extracellular matrix proteins, such as elastin and collagen (as well as fibrinogen), during neutrophil migration, damaging core lung structures - leading to the development of emphysema (Senior et al., 1977; Kao et al., 1988) and the amplification of neutrophilic inflammation. Clinically, an AAT serum level below 11 μM suggests an increased risk of developing emphysematous changes and airflow obstruction and has been termed the putative “at-risk threshold” (Wewers et al., 1987), generally accepted as the minimum level targeted by AAT augmentation therapy (Lucey et al., 1985).

The key sites of damage in COPD are the lung interstitium and the airways. Monitoring this process and its potential modulation presents a major challenge, although routine augmentation therapy reduces detectable inflammation in the airways (Stockley et al., 2002) and emphysema progression (Miravitlles et al., 2017). Studies have shown that neutrophil migration into the alveolar space is accompanied by fibrinogen fibrils (Burns et al., 2003). At such close proximity, degranulation releases NSPs which interacts with fibrinogen prior to inhibition by AAT, producing specific cleavage products that can recirculate into the systemic circulation via lymph drainage (Carter et al., 2011). Studies have shown that the specific NE cleavage product can be measured in the plasma, relates to the plasma AAT level consistent with a physiological threshold of protection at approximately 11 μM, and is reduced by augmentation therapy (Carter et al., 2011; Carter et al., 2015).

AATD plasma samples measured for NE- or PR3-specific activity footprints confirm that the levels are increased compared to healthy smoking volunteers and demonstrate different profiles between AAT genotypes that likely reflect the underlying pathophysiological processes (Newby et al., 2019). It was hypothesized that a proof-of-concept model could be developed, which would enable the assessment of the physiological and potential therapeutic impact on the proteinase/antiproteinase balance with specific characterization of the effect of AATD and its management. Proteinase activity footprints were used to describe the development and further validation of a novel neutrophil/fibrinogen-buffer model of footprint generation by uninhibited proteinase activity, termed the “proteinase/antiproteinase balance model.” The primary purpose of this model was to describe changes in proteinase activity related to inhibitor concentrations and support therapeutic applications, initially providing further data on the putative AAT “protective threshold” and potential efficacy of therapeutic inhibitors.

## 2 Materials and methods

### 2.1 Ethical statement

All research activities and biological materials used in this research study received ethical approvals listed in Sections 2.1.1 and 2.1.2. All subjects provided their written informed consent.

#### 2.1.1 Venous blood samples for peripheral neutrophil isolation

Healthy young volunteers (18–45 years old), non-smokers, were recruited for the study. Venous blood samples were collected from the volunteers in accordance with the University of Birmingham Research Ethics Committee under “Investigations of the ageing immune system” (ERN_12-1184). Recruitment criteria and demographics for the healthy young subjects are given in Supplementary Data S1.1 and Supplementary Table S1.

#### 2.1.2 Pooled plasma samples for the proteinase/antiproteinase model

Plasma for the AATD cohort was collected with ethical approval as part of the Antitrypsin Deficiency Assessment and Program for Treatment (ADAPT) (LREC Ref: 3359a). AATD subjects were of the PiZZ genotype. COPD plasma samples were collected under the study ethics of “Accelerated aging as a cause of disease pathogenesis, progression, and multi-morbidity in COPD” (REC 18/WM/0097). Healthy age-matched plasma was collected from volunteers under the ethics (REC 18/WM/0365) for the study “Neutrophil and Macrophage Phagocytosis in Alpha-1 Antitrypsin Deficiency.” Detailed cohort eligibility criteria are given in Supplementary Data S1.2; Supplementary Table S2.

### 2.2 Sample collection and plasma processing

Venous blood samples were collected in lithium heparin tubes (BD Vacutainer, United States) through venepuncture. The blood tubes were held at room temperature for 30 min and subsequently centrifuged at 1,000 × g for 10 min at room temperature. The plasma samples were harvested and stored in aliquots at −80°C.

### 2.3 Determination of AAT activity

AAT inhibitory activity was determined by titrating pure AAT (Athens Research and Technology) against porcine pancreatic elastase (PPE; Sigma-Aldrich) of known concentration and activity. The functional AAT activity was determined by the intercept of the *x*-axis determined by extrapolation (which reflects 1 to 1 molar equivalence). The pure AAT was calculated to be 83.0% active, and the active inhibitory concentration was used for all subsequent experiments.

### 2.4 Proteinase/antiproteinase balance model

#### 2.4.1 Isolation of neutrophils

Neutrophils from healthy young (HY) volunteers were isolated, as previously detailed by Jepsen and Skottun (1982); Grudzinska et al. (2023). In brief, whole blood was mixed with 2% dextran (Merck Life Science, United Kingdom; w/v in 0.154 M saline) for 30 min to allow buffy coat separation. Percoll (GE Healthcare, New York, United States) was diluted in 1.54 M of sterile saline at a ratio of 9:1 (v/v), from which 56% and 80% Percoll solutions were prepared (v/v, 0.154 M saline). The buffy coat was layered on top of the Percoll gradients and centrifuged at room temperature for 470 x g for 20 min without acceleration or brake. After centrifugation, the neutrophils were isolated and washed once in phosphate-buffered saline (PBS; Merck Life Science, United Kingdom) (250 × g, 10 min, full acceleration and break) before being re-suspended at a concentration of 2.5 × 10^6^ cells/mL in Roswell Park Memorial Institute (RPMI) 1640 Medium (with L-glutamine and sodium bicarbonate; Sigma-Aldrich, MO, United States) supplemented with penicillin–streptomycin (1% v/v; Sigma-Aldrich).

#### 2.4.2 Methodology for the proteinase/antiproteinase model

**FIGURE 1:**
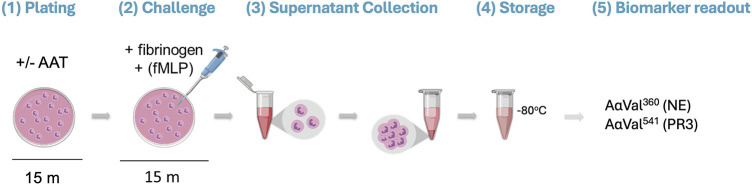
Visualization of the proteinase/antiproteinase balance model methodology. (1) Isolated healthy neutrophils are plated in a 24-well tissue culture plate at 2.5 million cells/mL in the presence or absence of AAT for 15 min at 37°C. (2) The substrate (fibrinogen; 550 nM) and stimulus (fMLP; 10 μM) are added to the neutrophils, followed by a 15-min incubation at 37°C. (3) Transfer of the cell suspension from the plate to a microtube. Supernatants are then generated from the treated cells by centrifugation (300 *x* g for 5 min). (4) The supernatant is transferred into a fresh microtube for storage at −80°C until required. (5) Supernatants are run on an in-house ELISA to detect specific fibrinogen cleavage products AαVal360 and AαVal541 to measure NE and PR3 activity, respectively. AAT, alpha-1 antitrypsin; fMLP, N-formylmethionine-leucyl-phenylalanine; NE, neutrophil elastase; PR3, proteinase 3.

Isolated healthy neutrophils (2.5 × 10^6^ cells/mL) in RPMI 1640 Medium (supplemented with L-glutamine, sodium bicarbonate, and 1% penicillin–streptomycin) were immediately plated in a 24-well tissue culture plate (Corning, NY, United States). The plated neutrophils were pre-incubated with or without AAT (of known molar activity) for 15 min at 37°C in doses based on plasma concentrations reported within the literature (Brantly et al., 1991; Kida et al., 1992; Kouchi et al., 1993; Sluis et al., 1994; Tsukishiro et al., 2005; Ferrarotti et al., 2012). Fibrinogen (550 nM, the substrate) and 10 μM of N-formylmethionine-leucyl-phenylalanine (fMLP; Merck Life Science, United Kingdom) were then added to the model for a further 15-min incubation at 37°C to facilitate neutrophil degranulation. After treatment, the neutrophils were removed from the plate and transferred into a sterile microtube. The supernatant was obtained by centrifugation (300 g for 5 min) and stored at −80°C until analysis (Figure 1).

### 2.5 Measuring proteinase activity footprints *via* the detection of AαVal^360^ and AαVal^541^

The proteinase activity footprints in the supernatants were determined *via* the generation of fibrinogen cleavage products AαVal^360^ (NE) and AαVal^541^ (PR3) *via* in-house inverse-fluorescence ELISAs, as previously described by Carter et al. (2011); Newby et al. (2019). In brief, the NE- or PR3-cleaved fibrinogen diluted in coating buffer (15 mM Na_2_CO_3_ and 35 mM NaHCO_3_, pH 9.6) was coated on a 96-well plate overnight at 4°C. The coated plates were washed in block buffer (Tris-buffered saline and 0.05% Tween 20, pH 7.4) and blocked with block buffer [Tris-buffered saline, 0.05% Tween 20, and 1% BSA (w/v), pH 7.4] ready for sample incubation. Two plates were run each time to control interplate variances. Standards (CJTSESSV for AαVal^360^ or COMLGEFV for AαVal^541^) were prepared by carrying out 2-fold serial dilutions. The standards or supernatant samples were incubated in duplicates with rabbit-specific anti-Aa antibodies overnight before being transferred to the fibrinogen-coated plates. After sample incubation, the plates were washed and incubated with a secondary anti-rabbit IgG Europium antibody (PerkinElmer, United States). The plates were subsequently washed, and the enhancement solution (PerkinElmer, United States) was added to each well in the dark. Finally, the plates were read at an excitation wavelength of 340 nm and an emission wavelength of 620 nm at 25°C on the BioTek Synergy 2 reader (BioTek, United States). The readings obtained from the standards were plotted against the standard concentration into a standard curve, from which the sample concentrations were obtained by interpolation.

### 2.6 Assay limits and coefficients of variation of the in-house proteinase activity assays for AαVal^541^ and AαVal^360^

Supernatants generated from 10 healthy individuals were used to evaluate assay variability. The analyte limits of the proteinase activity assay [i.e., the limit of blank (LoB), limit of detection (LoD), and limit of quantification (LoQ)] were defined and calculated using the adapted methodology published by Armbruster and Pry (2008), where LoB = mean of blank +1.645 (SD of the blank); LoD = LoB +1.645 (SD of the lowest analyte); LoQ ≥ LoD.

The mean value of the sample duplicates within the plate was used to calculate the intra-plate coefficient of variation (CV, %) using the formula (*SD/mean*) *100. The inter-plate CV was calculated by taking the sum of the sample CV from across two plates divided by the number of samples run. The AαVal^541^ assay had a LoB and LoD of 10.19 nM and 10.55 nM, respectively. The inter-assay CV was 42.9%; the intra-assay CV was 15.9%. The AαVal^360^ assay had a LoB and LoD of 0.86 nM and 1.72 nM, respectively. The inter-assay CV was 21.6%; the intra-assay CV was 13.7%.

### 2.7 Statistical analysis

Statistical analysis was performed using GraphPad Prism 8 software (GraphPad Software Inc., San Diego, CA). All experimental samples were run in duplicate, and the average value was determined as a result of each experiment. Replicate experiments are presented as the mean and standard error of the mean (SEM) or median and interquartile range (IQR) where appropriate. Statistical significance was assessed by either a Kruskal–Wallis test or mixed-effects analysis. The significance threshold was set at a *p*-value <0.05.

## 3 Results

### 3.1 Alpha-1 antitrypsin reduced proteinase footprint activity in the *in vitro* fibrinogen model

To assess how AAT concentrations observed in AATD affect proteinase activity in our model, we first incubated healthy neutrophils with pure AAT at concentrations reflecting the plasma AAT levels observed in AATD and normal healthy individuals. The mean percentage activity footprints (±SEM) of PR3 and NE are summarized in Table 1. The baseline was set at an inhibitor concentration of 0 μM as 100% enzyme footprint activity.

**TABLE 1:** Mean percentage activity footprint of PR3 and NE in the presence of AAT concentrations reflective of AATD. Here, 0 μM was set as 100% proteinase activity. Healthy young neutrophils from different donors (n = 4) were incubated with or without AAT and measured for PR3 and NE activity *via* the detection of AαVal^541^ and AαVal^360^ through an in-house ELISA. AαVal^541^ is a specific fibrinogen cleavage peptide for PR3. AαVal^360^ is a specific fibrinogen cleavage peptide for NE. Data are presented as the mean (±SEM). PR3, proteinase 3; NE, neutrophil elastase; AAT, alpha-1 antitrypsin; AATD, alpha-1 antitrypsin deficiency.

AAT concentration	0 μM (baseline)	3 μM	7 μM	10 μM	15 μM
PR3 (AαVal^541^)	100%	88.7% (±36.3)	57.14% (±31.76)	38.29% (±22.79)	37.76% (±24.34)
NE (AαVal^360^)	100%	70.05% (±9.0)	40.82% (±7.56)	41.99% (±10.77)	32.94% (±5.78)

**FIGURE 2:**
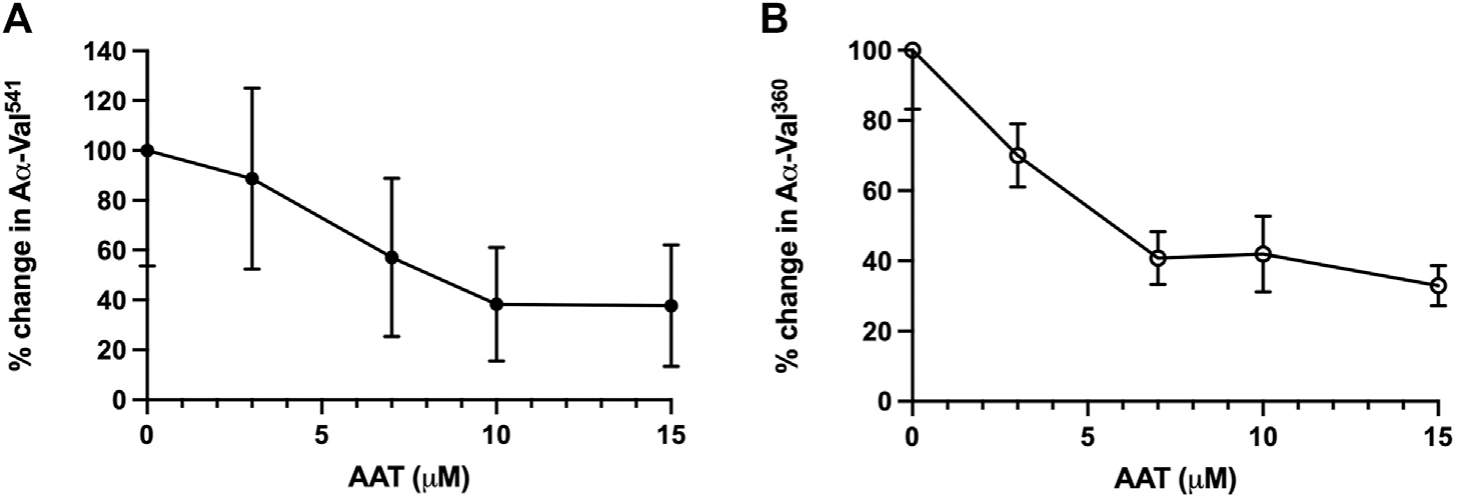
Incubation of neutrophils with AAT reduces the NSP activity footprint. Percentage activity of **(A)** AαVal541 (PR3 footprint) and **(B)** AαVal360 (NE footprint), following the incubation of isolated healthy neutrophils (2.5 × 10^6^/ml) in the presence of fMLP (10 μM) and uncleaved fibrinogen (550 nM) treated with increasing concentrations of AAT (0–15 µM). Neutrophils from different healthy donors (*n* = 4) were assessed for both AαVal^541^ and AαVal^360^ assays. Here, 0 μM was set as the 100% activity baseline. Data are presented in error bars as the mean ± SEM. AAT, alpha-1 antitrypsin; NSP, neutrophil serine proteinase; fMLP, N-formylmethionine-leucyl-phenylalanine; NE, neutrophil elastase; PR3, proteinase 3; SEM, standard error mean.

The level of fibrinogen cleavage products remained constant as the concentration of AAT decreased. However, at concentrations below 10 μM, an increase in the cleaved fibrinogen products generated was observed (Figure 2). The maximal inhibitory effect of AAT was achieved at concentrations above 10 μM, with the PR3 and NE footprint generation plateauing at 38.3% (±22.8) and 41.99% (±10.77), respectively. The maximal reduction in the proteinase footprint above 10 μM observed from the fibrinogen buffer model with AAT alone in the presence of activated neutrophils was supportive of the targeted treatment threshold of 11 μM for AAT augmentation therapy to provide maximum protection for local connective tissue from excess degradation in AATD (The Alpha-1-Antitrypsin Deficiency Registry Study Group, 1998).

### 3.2 Validation of the proteinase/antiprotienase model in plasma

We further validated this model in plasma to evaluate whether there are any differences in the footprint generation when using plasma from AATD, AAT-sufficient COPD, and healthy elderly individuals. The demographic characterization of the pooled plasma cohorts is given in Supplementary Table S1. Neutrophils isolated from HY donors were treated with pooled plasma from these three groups for 15 min at 37°C before being activated with fMLP (10 μM). The footprint activity generated after 15 min of stimulation was determined to be an increase compared that of the pooled plasma alone.

**FIGURE 3:**
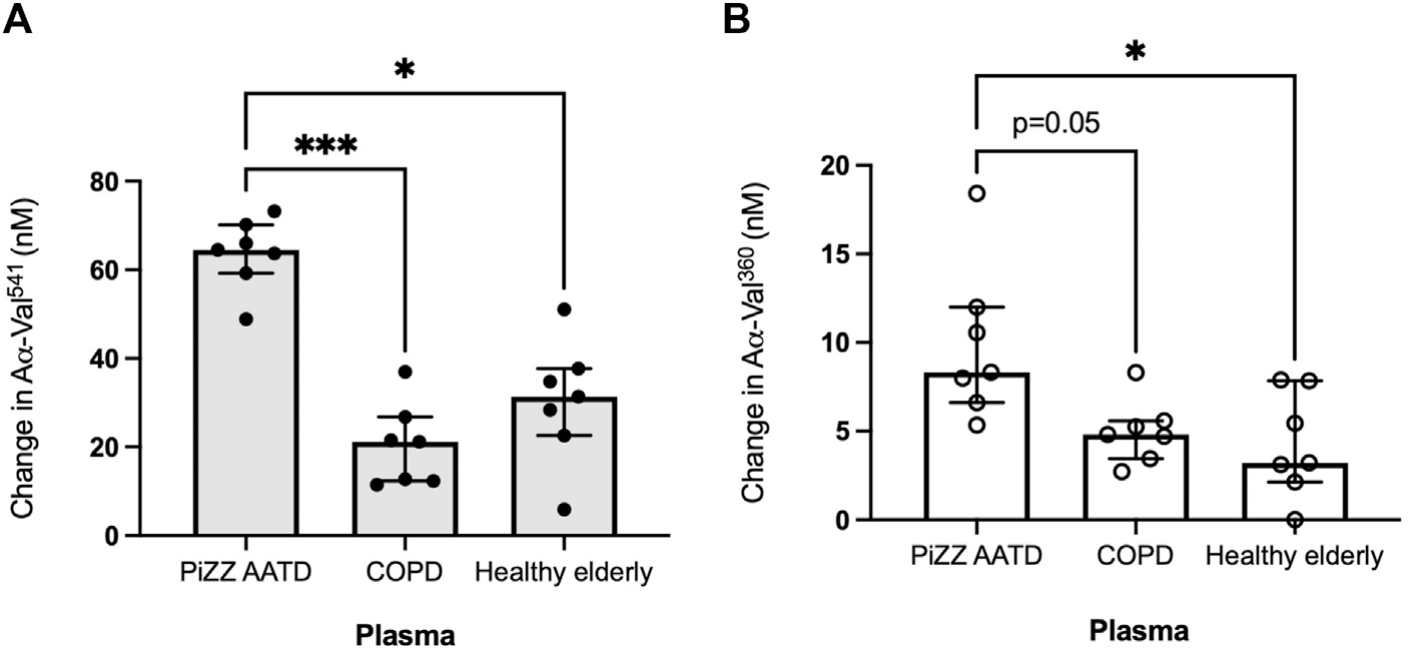
The proteinase footprint increases when neutrophils are incubated in AAT-deficient plasma. Median histograms of **(A)** PR3 and **(B)** NE footprints (*y*-axis) generated by neutrophils from healthy young subjects incubated in plasma from patients with non-deficient COPD or AATD or plasma from healthy elderly donors (*x*-axis). Healthy young neutrophils (*n* = 7) were incubated in plasma pooled from non-deficient COPD or AATD or healthy elderly donors. Subsequently, the supernatants were measured for AαVal^541^ and AαVal^360^. Each point is a single experiment. ● represents the PR3 activity footprint; o represents the NE activity footprint; error bars represent the median ± IQR; * = *p* < 0.05 and *** = *p* ≤ 0.001 determined by the Kruskal–Wallis test with multiple comparisons. AAT, alpha-1 antitrypsin; AATD, alpha-1 antitrypsin deficiency; COPD, chronic obstructive pulmonary disease; NE, neutrophil elastase; PR3, proteinase 3; IQR, interquartile range.

When incubated with healthy elderly plasma, HY neutrophils generated a median increase of 31.38 nM (IQR 22.6–37.7) and 4.3 nM (2.9–7.9) for PR3 and NE activity footprints, respectively (Figure 3). This change was similar when non-deficient COPD patient plasma was used, with a median PR3 footprint generation of 21.2 nM (12.3–26.7) (*p* = 0.788) and 4.8 nM (3.4–5.6) for the NE footprint (*p* > 0.999, Kruskal–Wallis test). However, when incubated in AATD patient plasma, the activity footprint generated was significantly greater at 64.5 nM (59.2–70.2) for PR3 (*versus* healthy plasma *p* < 0.05 and COPD plasma *p* < 0.001) and 8.3 nM (6.6–12.0) for NE (*versus* healthy *p* < 0.05 *versus* COPD *p* = 0.05).

The data presented indicated the effect of ambient AAT concentrations in plasma. The data demonstrate that neutrophils obtained from healthy individuals can generate footprints even when incubated in plasma, and that this is enhanced when plasma from subjects with AATD is used. Again, these data are consistent with the effect of low AAT levels on the generation of specific enzyme footprints in the presence of activated neutrophils.

### 3.3 Treatment of neutrophils in an *in vitro* model with MPH966 reduced proteinase activity

MPH966 (also known as alvelestat or AZD9668, Mereo BioPharma), a highly selective reversible inhibitor of NE (Stevens et al., 2011), was added to the stimulated fibrinogen model to investigate the inhibitory effect of MPH966 on proteinase fibrinogen footprint generation. MPH966 was added to the model (with neutrophils from HY controls) in doses reflecting the therapeutically relevant concentrations previously reported in the literature (10 nM–700 nM) (Stevens et al., 2011; Vogelmeier et al., 2012; Gunawardena et al., 2013). Each experiment accounted for different cell preparations by expressing control over enzymatic footprint generation as 100%. The effect of MPH966 was determined for both PR3- and NE-generated footprints, and the results were reported as the remaining percentage activity (±SEM) for AαVal^360^ or AαVal^541^ generation, compared to the baseline control.

**FIGURE 4:**
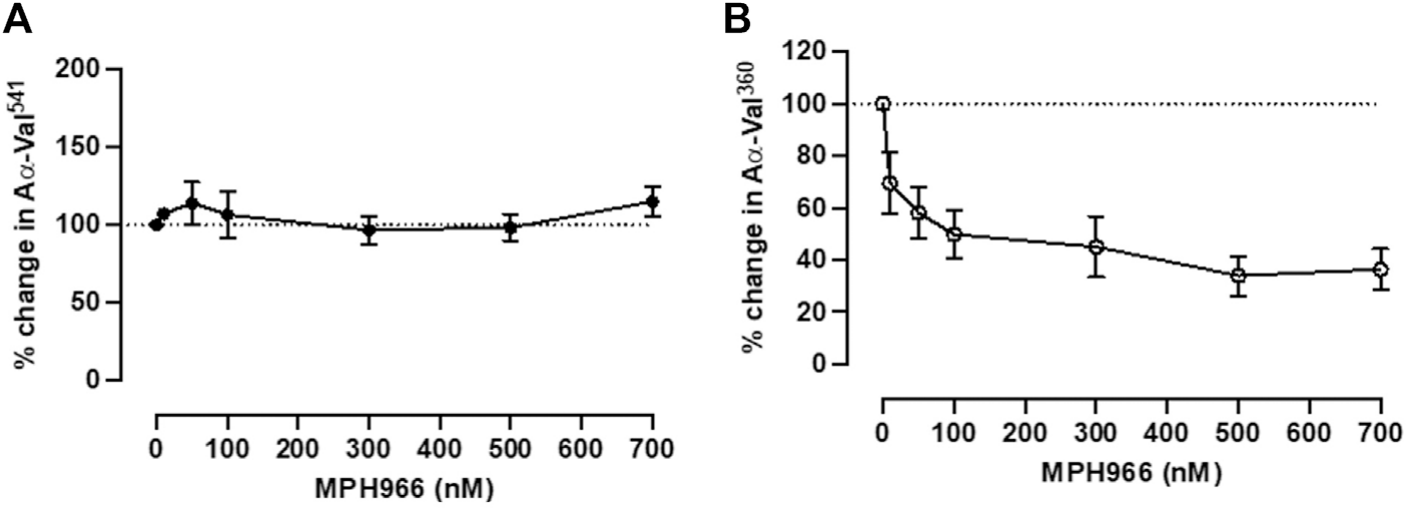
Neutrophils treated with MPH966 alone reduced the NE but not the PR3 activity footprint. The proteinase footprint expressed as % of the control (baseline set at 100%, *y*-axis) produced by fMLP-stimulated neutrophils in the presence of increasing concentrations (0–700 nM) of MPH966 (*x*-axis); percentage activity in the **(A)** PR3 footprint and **(B)** NE footprint. Neutrophils from different healthy donors (*n* = 7) were assessed for both AαVal^541^ and AαVal^360^ assays. Dotted line at 100%; ● represents the PR3 activity footprint; o represents the NE activity footprint; error bars represent the mean ± SEM. fMLP, N-formylmethionine-leucyl-phenylalanine; AAT, alpha-1 antitrypsin; AATD, alpha-1 antitrypsin deficiency; COPD, chronic obstructive pulmonary disease; NE, neutrophil elastase; PR3, proteinase 3; MPH966, selective NE inhibitor; SEM, standard error mean.

Incubation with MPH966 reduced the NE footprint at all assessed doses but not the PR3 footprint as expected (Stevens et al., 2011) (Figure 4). The PR3 activity footprint remained between 92.0% (±16.5%) and 128.0% (±18.9%) for all drug concentrations tested (Figure 4A). The activity footprint for NE decreased to 62.4% (±10.8%) at 10 nM and continued to decrease with increasing inhibitor concentrations until plateauing at the pharmacological dose of 300 nM [32.9% (±11.4%), Figure 4B]. MPH966 alone reduced the generation of the NE footprint at a lower molar concentration than observed for AAT (Figure 2B; Figure 4B). This would imply that MPH966 can inhibit some of the NE that is not freely accessible to the larger AAT molecule, such as targeting membrane-bound or intracellular NE. However, the exact mechanism is currently unknown.

### 3.4 Effect of MPH966 on PR3 and NE activity footprints in the presence of increasing AAT concentrations

As it has been previously established that AAT preferentially binds to NE compared to PR3 (reflected by the relative association rate constants) (Korkmaz et al., 2005; Sinden et al., 2015), we also investigated whether the highly NE-selective inhibitor MPH966 has an additive inhibitory effect on NE footprint generation while enabling a greater proportion of AAT to inhibit PR3 footprint generation. To test this, we concurrently introduced MPH966 to the *in vitro* model at the proposed therapeutic concentration (300 nM) with or without AAT at concentrations covering the deficient AAT levels observed in patients without or receiving AAT augmentation therapy (0, 5, 10, and 15 μM).

**FIGURE 5:**
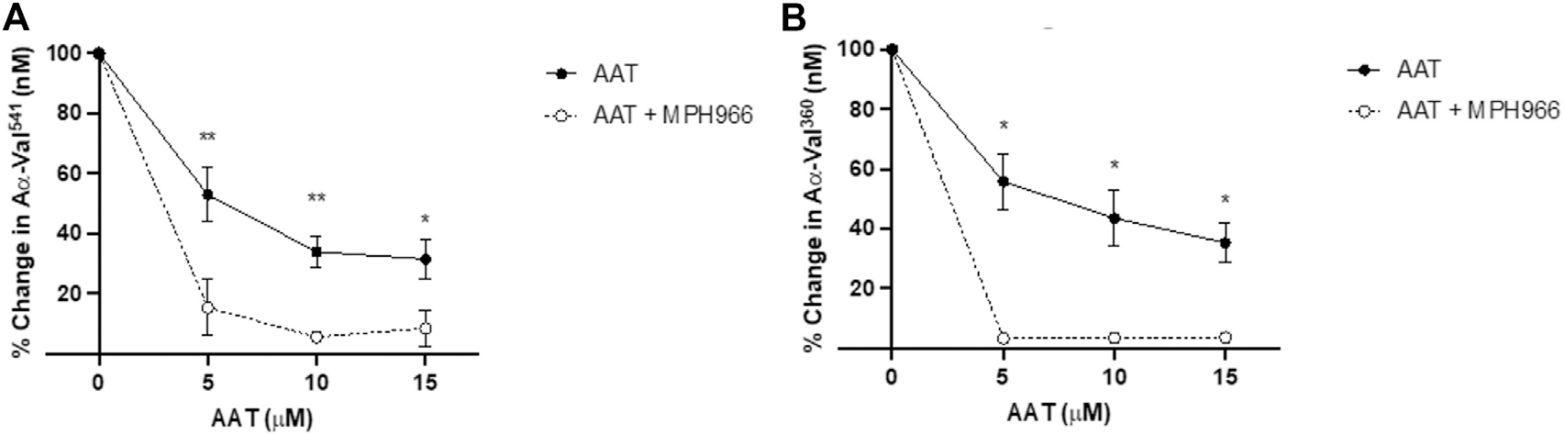
Comparison of proteinase activity footprint following the application of AAT with and without MPH966. Percentage change in the **(A)** PR3 and **(B)** NE activity footprint (*y*-axis) from fMLP-stimulated neutrophils (2.5 × 10^6^/ml) in a fibrinogen buffer (550 nM) inhibited with AAT (*x*-axis) at 5, 10, or 15 µM (with solid line), compared to fMLP-stimulated neutrophils in a fibrinogen buffer with MPH966 (300 nM) and AAT at 5, 10, or 15 µM (with dotted line). Neutrophils from different healthy donors (*n* = 4) were assessed for both AαVal^541^ and AαVal^360^ assays. ● represents the PR3 activity footprint; o represents the NE activity footprint. Error bars indicate the mean±SEM. * *p* < 0.05 and ** = *p* ≤ 0.01 determined by mixed-effects analysis. fMLP, N-formylmethionine-leucyl-phenylalanine; AAT, alpha-1 antitrypsin; AATD, alpha-1 antitrypsin deficiency; COPD, chronic obstructive pulmonary disease; NE, neutrophil elastase; PR3, proteinase 3; SEM, standard error mean.

Neutrophils treated with MPH966 in conjunction with and without various concentrations of AAT led to a further decrease in both NE and PR3 activity footprints compared to MPH966 alone (Figure 5). From an uninhibited control as baseline (100% active), the addition of MPH966 to a sample with 5 μM AAT compared to inhibition with 5 μM AAT alone significantly reduced the PR3 activity footprint from 52.7% (SEM ±9.0) to 15.4% (±9.5) (*p* = 0.001; mixed-effect analysis; Figure 5A) and the NE activity footprint from 57.1% (±9.3) to 3.2% (±0.7) (*p* = 0.009; Figure 5B).

With 10 μM of AAT, MPH966 reduced the PR3 activity footprint from 42.0% (±5.3) to 5.6% (±1.7) (*p* < 0.01; mixed-effects analysis) and the NE activity footprint from 38.3% (±9.5) to 3.4% ( ± 0.8) (*p* < 0.05). With 15 μM of AAT, the PR3 activity footprint was reduced from 32.9% (±6.7) to 8.5% (±6.0) (*p* < 0.05), and the NE activity footprint was reduced from 37.8% (±6.6) for AAT alone to 3.4% (±0.6; *p* < 0.05).

The addition of averaged pathophysiological concentrations of AAT and therapeutic concentrations of MPH966 to the model produced greater inhibition of proteolytic footprint generation by both NE and PR3. The observed effect of MPH966 was consistent with the partitioning of NSPs into AAT previously described by Sinden et al. (2015).

## 4 Discussion

We established an *in vitro* model to mimic the proteinase/antiproteinase balance in the presence of activated neutrophils and a target substrate generating specific NSP products measurable *in vivo*. The model would allow the assessment of the generation of NE and PR3 footprint markers in the presence of natural or pharmacological inhibitors for interpreting relevant *in vivo* data. The use of stimulated neutrophils and cleavage proteinase-generated fibrinogen cleavage products (AαVal^360^ and AαVal^541^) are considered useful biomarkers already documented as having measurable effects *in vivo*, providing early readouts as proof of concept for putative clinical efficacy. The changes in the enzyme-specific footprints were validated in plasma-treated neutrophils, reflecting the concentrations of AAT in the disease and providing further support for the model.

Measuring NE and PR3 activities as a treatment outcome is particularly important when investigating the treatment effects of chronic neutrophilic diseases, such as AATD, where these enzymes are considered primary drivers of the disease process. To date, assessing *in vivo* NE and PR3 activity has been made difficult by the broad range of shared substrates and the generation of non-specific cleavage products (Viglio et al., 2021). We previously showed the specificity and reliability of the fibrinogenic peptides AαVal^360^ and AαVal^541^ as markers of NE and PR3, respectively (Carter et al., 2011; Newby et al., 2019). Both of these enzymes have been implicated in the pathophysiology of AATD-mediated lung disease (Stockley, 2020). Thus, the assessment of both NE and PR3 activity would allow the investigation of drug specificity and any synergistic effect, especially as evidence suggests that PR3 may play a greater role in driving emphysema generation than that attributed historically to NE alone (Korkmaz et al., 2005; Sinden et al., 2015). Our *in vitro* model captured the enzymatic footprint changes consistent with clinical observations in patients and provides a quick and reproducible way to assess the cellular response effects of putative therapeutic compounds for the NSP axis and AAT at levels consistent with AATD before and during augmentation therapy.

The current model is limited to assessing only NE and PR3 footprints and does not evaluate the potential impact of cathepsin G, which has also been reported to contribute to elastin degradation, at least in a cigarette smoke-induced animal model of emphysema (Guyot et al., 2014). Current literature concerning the direct involvement of cathepsin G in AATD remains scarce (Lucey et al., 1985; Guyot et al., 2014). A similar fibrinogen-specific cleavage can be generated to assess the possible cathepsin G contribution to the overall pathophysiology of AATD. However, at present, the model provides a way to assess therapeutics that can primarily affect NE or PR3 activity footprints *via* upstream neutrophil activation and degranulation, as well as direct inhibition. This novel *in vitro* model provides a quick, efficient, and cheaper first step to investigate potential therapeutics for animal models with markers specific to humans for subsequent early phases of drug development.

Based on the pharmacokinetic profile of MPH966, it was reported that a clinically meaningful effect requires a plasma concentration greater than 300 nM of MPH966 (Gunawardena et al., 2013) and that a minimum dosing regimen of at least 240 mg twice daily (*bid*) is required to achieve this in humans (Parkin, 2022). The observed maximal inhibitory effect of MPH966 on proteinase footprints from our model at this clinical dose (300 nM) implied that the model provides supporting evidence for determining the effective dose for phase I/II trials, enabling informed decisions on suitability and dosing for phase III clinical efficacy studies. This was corroborated in the recently completed 12-week clinical trial investigating the effects of MPH966 in patients with AATD (NCT03636347). The investigators reported a significant, consistent reduction in the NE footprint (AαVal^360^) compared to baseline (−22.7%, *p* = 0.004) in the high-dose arm (240 mg *bid*) while not observed in the low-dose arm (120 mg *bid*) (Stockley et al., 2023).

### 4.1 Limitation of the model

It has been established that circulating neutrophils are functionally different as a result of healthy aging (Hazeldine et al., 2014) and in chronic neutrophilic diseases such as COPD (Kapellos et al., 2023) and AATD (Hawkins et al., 2021). Our optimized model using neutrophils from healthy young volunteers may not reflect responses specific to the neutrophils of aging populations. For instance, AATD neutrophils have been reported to be inherently primed (McEnery et al., 2022). Evidence of increased reactive oxidative species and other functional neutrophil outputs that may reflect increased NSP activation has been observed in more elderly subjects (Hawkins et al., 2021). It is possible that AATD neutrophils are likely to degranulate more readily, which could influence the subsequent treatment effect of any experimental compounds tested. A comparison of the current data with that using AATD patient neutrophils would indicate whether such a difference *in vitro* might be expected *in vivo*. Furthermore, the Z-AAT protein from the most severe form of AATD is known to inhibit NSPs less avidly than normally folded AAT (M-AAT) (Korkmaz et al., 2005; Sinden et al., 2015), and spontaneous polymerization of the Z protein is also known to activate neutrophils, adding further complexity (McEnery et al., 2022) to the physiological effects in patients.

Our model currently employs M-AAT to simulate pathological concentrations observed in AATD, including those on augmentation who have a combination of the Z and infused M-AAT proteins.

The *in vitro* model can be easily adapted to investigate the potential effects of the Z and M-AAT protein combinations and account for any inherently different cellular responses relevant to the predicted pharmacological outcomes, as well as the effects of persisting Z-AAT on neutrophil activation.

The activation of neutrophils in chronic inflammatory disease is also dependent on a variety of chemokines other than the standard fMLP used here. However, again, the model permits other relevant cytokines from the disease *milieu* to be included as indicated. Whether such factors influence footprint generation *in vivo* remains speculative in the absence of specific clinical trials.

## 5 Conclusion

We present an *in vitro* model of the proteinase/antiproteinase balance in the presence of activated neutrophils and a susceptible substrate. The data demonstrated the role of AAT in suppressing the destructive actions of NE and PR3 in AATD and illustrated how a reduction in AAT results in increased pericellular activity of proteinases. The data further provided supportive evidence of a threshold below which neutrophilic activity is enhanced and a putative dose-ranging template for new compounds to be assessed for equivalence or additive impact on AAT for therapeutic strategies. Finally, the data presented suggested that MPH966 may function as an effective inhibitor of excessive pericellular proteinase activity at the low concentrations observed in pharmacokinetic studies, and together with AAT data, this suggests that the inhibitor has an additive effect. The evidence supports its further development in phase III trials of AATD alone or in combination with AAT augmentation therapy.

## Data Availability

The original contributions presented in the study are included in the article/Supplementary Material; further inquiries can be directed to the corresponding author.
