# Supplementary material for: A novel in vitro cell model of the proteinase/antiproteinase balance observed in alpha-1 antitrypsin deficiency
Source: Front Pharmacol. 2024 Jul 1;15:1421598. doi: 10.3389/fphar.2024.1421598 (PMC11250411; doi:10.3389/fphar.2024.1421598)
Supplement: Supplementary file 1 [file DataSheet1.docx]

Supplementary Material

# Supplementary Data

*1.1 Inclusion and exclusion criteria for cohort selection for subjects providing neutrophils.*

Healthy young adults (< 45 years old) with no evidence of clinical disease or symptoms of current or recent respiratory disease.

*1.2 Inclusion and exclusion criteria for cohort selection for subjects providing plasma.*

1. Healthy older adults (≥ 45 years old) with no evidence of clinical disease or symptoms of current or recent respiratory disease.
2. AATD: A confirmed PiZZ genotype and stable disease (no exacerbation in the previous 6 weeks). Patient is not eligible if any of the following applied: evidence of lung cancer, hematological malignancy, previous lung resection, treatment with regular oral prednisolone or immunosuppressive medication.
3. Non-deficient COPD: patient (≥ 40 years old) diagnosed with stable proven COPD with at least a 10 pack-year smoking history. Patient is not eligible if any of the following applied: a diagnosis of any alternative lung disease (i.e. asthma, interstitial lung disease or lung cancer), or recent exacerbation of disease (within the previous 6 weeks).

# Supplementary Tables

**Supplementary table 1. Demographics of healthy subjects providing neutrophils**. Age, sex, and smoking status of healthy young donors (aged < 45 years) whose neutrophils were used to generate proteinase activity footprints. SD could not be calculated for pack year history as only one subject recruited had a history of smoking. *SD: Standard deviation.*

|  | **Healthy young adults** |
| --- | --- |
| Total, n | 11 |
| Age, mean (range) | 33.5 (22-37) |
| Sex, male (%) | 8 (73) |
| Current smokers (%) | 0 (0) |
| Ex-smokers (%) | 1 (1) |
| Mean pack year history (± SD) | 5.0 (N/A) |

**Supplementary table 2. Demographics of subjects providing plasma pools**. Age, sex, lung function (FEV_1_ % predicted), and smoking status of donors whose plasma were used to generate a pool for experimentation are listed. Three plasma pools were generated. Namely, stable AATD (of PiZZ genotype), stable non-deficient COPD, and healthy older adults. *FEV_1_: Forced expiratory volume in 1 second, AATD: alpha-1 antitrypsin deficiency, COPD: chronic obstructive pulmonary disease, SD: Standard deviation.*

|  | **AATD** | **Non-deficient COPD** | **Healthy older adults** |
| --- | --- | --- | --- |
| Total, n | 13 | 20 | 20 |
| Age, mean (range) | 60.9 (48-81) | 70.8 (60-83) | 71.5 (59-95) |
| Sex, male (%) | 10 (77) | 14 (70) | 9 (45) |
| FEV_1_ % predicted, mean (± SD) | 47.2 (± 22.0) | 54.0 (±22.3) | 116.5 (±19.1) |
| Current smokers (%) | 1 (7) | 2 (10) | 2 (10) |
| Ex-smokers (%) | 8 (62) | 18 (90) | 7 (35) |
| Mean pack year history (± SD) | 18.0 (± 11.3) | 54.1 (± 53.7) | 7.2 (± 11.7) |
